# Counteracting the Toxic Hidroarsenicism Effects upon Reproductive-Physiological Outcomes in Male Goats: The Selenium and Vitamin-E Mitigation Approach

**DOI:** 10.3390/ani13132055

**Published:** 2023-06-21

**Authors:** Natalia B. Ortega-Morales, Jose A. Cueto-Wong, Leonardo I. Velez-Monroy, Adan U. Chavez-Solis, Eutiquio Barrientos-Juarez, Jesús Jaime Duarte-Sustaita, Francisco G. Veliz-Deras, Guadalupe Calderon-Leyva, Javier Moran-Martinez

**Affiliations:** 1 Autonomous Agrarian University Antonio Narro, Torreón 27054, Coahuila, Mexico; nabel_87@hotmail.com; 2 National Institute of Forest, Agricultural and Livestock Research, La Laguna Experimental Field, Matamoros 27440, Coahuila, Mexico; cueto.jose@inifap.gob.mx (J.A.C.-W.); velmonli@yahoo.com.mx (L.I.V.-M.); chavez.adan@inifap.gob.mx (A.U.C.-S.); 3 National Institute of Forestry, Agricultural and Livestock Research, Campo Experimental Aldama, Juan Aldama 32910, Chihuahua, Mexico; barrientos.eutiquio@gmail.com; 4 Faculty of Health Sciences, Juárez University of the State of Durango, Durango 34120, Durango, Mexico; qfb.jaimeduarte@gmail.com; 5 Department of Veterinary Sciences, Autonomous Agrarian University Antonio Narro, Torreón 27054, Coahuila, Mexico; velizderas@gmail.com; 6 Department of Animal Production, Autonomous Agrarian University Antonio Narro, Torreón 27054, Coahuila, Mexico; gcalderon06@hotmail.com; 7 Department of Cell Biology and Ultrastructure, Faculty of Medicine, Autonomous University of Coahuila, Torreón 27000, Coahuila, Mexico

**Keywords:** arsenic, male goats, physiological variables, selenium, vitamin-E, seminal quality

## Abstract

**Simple Summary:**

This study demonstrates that treatment with selenium and vitamin-E reduced the damage caused by sodium arsenite in sperm quality in goats. The present investigation is a reference to propose the use of these antioxidants to reduce damage caused by arsenic in the animal organisms in places where groundwater contaminated with arsenic is used as drinking water for animals.

**Abstract:**

This study was to evaluate whether selenium and vitamin-E counteract the toxic effects of arsenic on reproductive aspects and physiological conditions of male goats. Male goats [Criollo, *n* = 20, 4–5 yr-old, 72 kg live weight (LW)] were distributed in homogeneous groups (*n* = 5), and received during 12 weeks: (1) Sodium arsenite 2 mg/kg of LW/day (AG, LW = 69 kg); (2) Sodium selenite 6 mg + vitamin-E 420 I.U. every 14 days during the 12 weeks (SG, LW = 68 kg); (3) Both AG and SG treatments (ASG, LW = 77 kg); and (4) 1 mL of physiological solution every 14 days during the 12 weeks (CG, LW = 72 kg). The animals had access to water from a well with a concentration of 35 μg/L of arsenic. The SG had the highest percentage of sperm viability (80.6%) followed by the CG, ASG (74.7; *p* > 0.05), and AG (64.3; *p* ≤ 0.05). The ASG and SG had a lower heart rate as compared to the CG and AG (58.8, 58 vs. 65.4, 63.5; respectively, *p* ≤ 0.05). The CG and SG showed a lower respiratory rate than the AG and ASG (19.2, 18.7 vs. 22.1, 21.0, respectively; *p* ≤ 0.05). Selenium and vitamin-E were efficient in reducing the damage caused by sodium arsenite in sperm quality and maintaining heart and respiratory rates and increases in odor in male goats.

## 1. Introduction

Arsenic is present in the environment in soil, water, and air; the compounds of arsenic are odorless and lack a special flavor, which is why it is impossible to detect their presence in food, water, or air [1]. The presence of arsenic in drinking water (hydroarsenicism) is a health problem of global importance [2]; there are reports of chronic poisoning from exposure to high concentrations of arsenic in water mainly in countries of Asia and Latin America [1,3].

In Latin America, the permitted limit of arsenic in drinking water has seen a gradual approach in which limits have been established according to the affordable technology in each country. Nowadays, the tested limits can reach 10 μg/L as recommended by the WHO [4]; in the specific case of Mexico, it is 25 μg L^−1^ [5]. However, the maximum permissible limits for animal consumption are not yet well established; therefore different values have been reported, including 25, 50 and 200 μg L^−1^ [6,7,8]. Similarly, various studies have been carried out to assess arsenic toxicity in animals such as rodents, cattle, sheep, and goats [9,10,11].

In goats, it has been found that consuming water contaminated with arsenic can cause damage to the body and the physiological condition [12], because exposure to arsenic results in its deposition in different body tissues such as the heart, muscles, kidneys and liver of animals, which produces different toxic effects in these tissues [13]. On the other hand, there are reports that arsenic also causes damage at the reproductive level; in fact, damage has been found in the epididymis and vas deferens [14], as well as in sperm quality [11,15,16]. These alterations caused by the high levels of arsenic in the animal’s organism can be attributed to the oxidative stress it exerts, as it can damage DNA, leading to a cascade of events, some of which can be adverse. Such effects can be coated or diminished by selenium, due to its antioxidant action, which can contribute to diminishing, by various mechanisms, the toxic effects produced by arsenic. The beneficial effect of selenium can be favored by vitamin-E, which acts as the first line of defense against oxidative stress [17]. Selenium and vitamin-E are two antioxidants that act synergistically and must be administered together [18]; in addition, both act at the reproductive tissue level, and during spermatogenesis, improving semen quality [11,19]. On the other hand, it has been shown that selenium in inadequate doses (deficiency or excess) can deleteriously alter spermatogenesis in species such as mice and broilers, among others. In these studies, part of the reproductive oxidative-stress process due to selenium was observed [20,21,22]. On the other hand, physiological alterations due to exposure to arsenic in goats or other mammals have been little analyzed. Some studies carried out with arsenic exposure in rats show an important effect on the vascular system, endothelial damage, and hypertension, as well as damage to the respiratory system [23,24,25]. Other physiological variables in male goats such as odor, respiratory rate and temperature have not been associated with arsenic exposure. In many parts of the world, both hidroarsenicism and goat production are constants in arid zones, as occurs in the Comarca Lagunera, Mexico. Based on the previous information, we aimed to determine if selenium and vitamin-E can counteract the toxic effects of arsenic on reproductive aspects and physiological conditions in goat males.

## 2. Materials and Methods

### 2.1. Ethics Statement

All the methods and management of the experimental units used in this study were in strict accordance with accepted guidelines for ethical use, care and welfare of animals in research at international [26] and national [27] levels, and in accordance with the authorization of the Institutional Committee for the Care and Use of Laboratory Animals (CICUAL) of the Faculty of Medicine of the Autonomous University of Coahuila (Reference number: 01a-01-18).

### 2.2. Location, Experimental Animals, and Treatments

The present study was carried out in the La Laguna Experimental Center, dependent on the National Institute for Forestry, Agriculture and Livestock Research (INIFAP), located in Matamoros, Coahuila, Mexico (25° N, 103° W). Criollo male goats, clinically healthy and sexually active (*n* = 20, 4–5 yr-old, 72 ± 5 kg live weight (LW)), were considered. The diet consisted of a mixture of alfalfa, fodder corn and concentrate (18% PC), with ad libitum access in the feeder; the bucks had access to water from a well at the Center, with an arsenic concentration of 35 ± 5 μg/L. The bucks were distributed into four homogeneous groups (*n* = 5, each) based on their LW and age, and during the 12 weeks of the experimental period they received: (1) Sodium arsenite 2 mg/kg LW/day, orally. (AG, LW = 69 ± 1.1 kg); (2) Sodium selenite 6 mg + vitamin-E 420 I.U. every 14 d, applied by subcutaneous injection (SC); (SG, LW = 68 ± 1.5 kg); (3) AG + SG treatments (ASG, LW = 77 ± 7.4 kg); and (4) Physiological saline solution 1 mL, SC, every 14 days (CG, LW = 72 ± 4 kg). The arbitrary scale used for the protocol of the evaluation of some reproductive and physiological variables (1–3) for male goats is the one established and standardized in the National Institute of Forestry, Agriculture and Livestock Research (INIFAP) [28].

### 2.3. Response Variables

**Figure 1:**
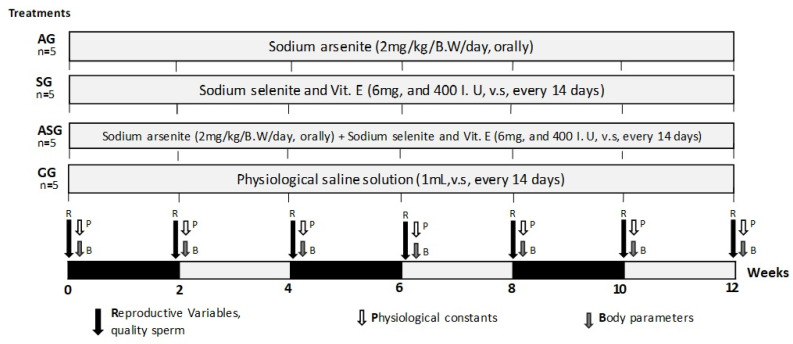
Distribution of groups (*n* = 20), application of treatments and variables evaluated in male goats treated with sodium arsenite (AG), sodium selenite and vitamin E (SG), arsenite + sodium selenite and vitamin E (ASG) and physiological solution (CG) for 12 weeks (Total number of animals = 20).

The study was conducted during the breeding season and the response variables considered sperm quality, physiological constants, and body parameters, which were collected every 14 days for 12-weeks (Figure 1).

### 2.4. Reproductive Parameters

Scrotal circumference (cm), was measured with a flexible tape at the widest part of the testes; odor intensity (0–3) was recorded by smelling above the base of the horns at a distance of 8–10 cm, as described by Walken-Brown et al. (1994) [29]; latancy to ejaculation: to evaluate this variable, the bucks were exposed to a female induced to estrus by injecting estradiol benzoate (100 g twice a week). The male had to ejaculate within three minutes of exposure to the goat [30]; sperm quality: semen was extracted from each male goat, exposed to an estrus-phase female goat, through an artificial vagina for sheep, at a temperature of 37 °C. The extracted semen was immediately transported to the laboratory and placed in a water bath to maintain it at a temperature of 37 °C, in order to proceed with its analysis. Ejaculate volume (mL) was determined directly from a conical extraction tube, located at the end of the artificial vagina; sperm motility (scale 1–3) was assessed by placing a drop of semen in an object holder, and was observed in a microscope with optical phase contrast (400×), where movement was observed in circular waves (scale 3) or absence of mobility (scale 1); sperm viability (%) was evaluated by placing a drop of semen and a drop of eosin/fuchsin staining at one end of the slide; they were mixed and immediately evaluated with a microscope (1000×) and by counting 200 sperms differentiating between the number of live (unstained) and dead (stained) sperms in order to calculate the percentage of viability; the spermatic concentration (10^6^/mL) was automatically measured with an absorbance-based photometer (SpermaCue, minitube, model SDM1); the color (scale 1–3) was determined visually, distinguishing between a creamy color (3 = highest quality), white (2 = intermediate quality) and yellow (1 = low quality).

### 2.5. Physiological Constants

Heart rate (bpm), was considered, counting beats per minute using a stethoscope; the respiratory rate (rpm) was evaluated by counting the breaths per minute with the help of a stopwatch; rumen movement (scale 1–3) was determined by ventral palpation for one minute, recording the speed of movement and classifying it on a scale from 1 (when movement was almost null) to 3 (when there was a high frequency of movement); and body temperature (°C) was measured with a digital thermometer directly from the anus.

### 2.6. Body Parameters

Live weight (LW, kg) was measured with a pen scale with a capacity of 100 kg and with a precision range of 0.5 kg; body condition score (BCS, scale from 1 to 3) was determined by dorsal palpation using a scale from 1 to 3 (1 reflecting very skinny animals, and 3 for obese animals).

### 2.7. Statistical Analysis

The data obtained from the reproductive aspects, physiological constants and body parameters were analyzed through a two-way ANOVA of repeated means; when significant values were obtained (*p* ≤ 0.05), a comparison of means was made, using post-hoc tests (Tukey). All analyses were performed using the SPSS statistical package [31].

## 3. Results

**Figure 2:**
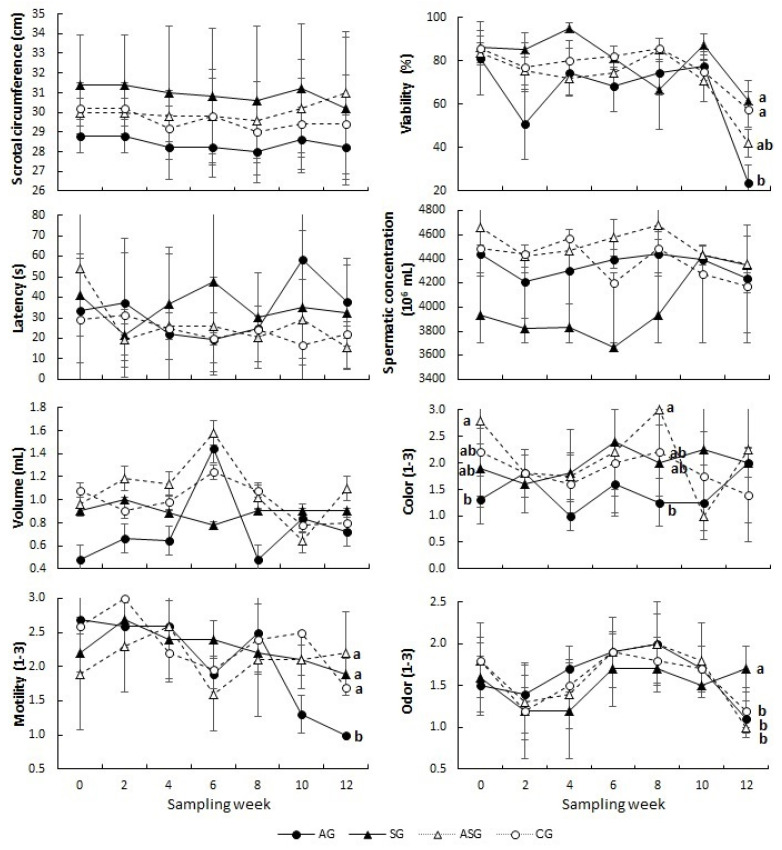
Reproductive parameters measured across time in the male goats treated with sodium arsenite (black circles solid lines; AG, *n* = 5), sodium selenite and vitamin E (black triangles solid lines; SG, *n* = 5), arsenite + sodium selenite and vitamin E (black circles lines dashed; ASG, *n* = 5), and physiological solution (black circles dashed lines; CG, *n* = 5), for 12 weeks. ^a, b^ Values with different literal difference (*p* ≤ 0.05). Mean values (±SEM).

Table 1 shows the results of the reproductive variables of the male goats treated with arsenic, selenium, arsenic plus selenium, and a physiological saline solution. No differences among treatments occurred regarding either time or the interaction of treatment × time in the scrotal circumference (29.75 ± 2.03; *p* > 0.05). In contrast, odor differed across time (*p* ≤ 0.05); the SG generated the stronger odor at week 12 compared to CG, AG and ASG (1.7 ± 0.27 vs. 1.2 ± 0.27, 1.1 ± 0.22 and 1.0 ± 0; respectively, *p* ≤ 0.05; Figure 2). Regarding semen quality, the highest percentage of live sperms (80.6 ± 19.9) was observed in the SG, followed by the CG and ASG (74.7 ± 17.7; *p* > 0.05), the lowest percentage was detected in the AG group (64.3 ± 29.3; *p* ≤ 0.05).

In the variables of volume, sperm motility and color, statistical differences across time were observed. In the volume and sperm motility variables, the SG stood out at week 12, and in color at week 8 in the SG (Figure 2). Regarding the ejaculation latency variable, no significant differences were found between the treatments (*p* > 0.05); these variations are observed in Table 1 and Figure 1. The bucks treated with selenium started with the lowest sperm concentration (3933.20 ± 1196.1), 13% lower compared to the other groups (AG, ASG and CG; 4530.8 ± 290.8). However, across the supplementation it can be observed that from the 6th week the sperm concentration increased in the SG group, finally exceeding the AG and CG groups at the end of the study; however, no significant statistical differences were observed (*p* > 0.05, Figure 2).

**Figure 3:**
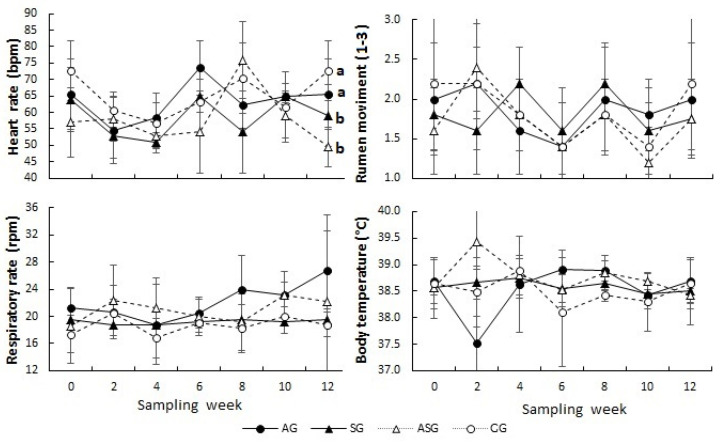
Physiological constants measured across time in the male goats treated with sodium arsenite (black circles solid lines; AG, *n* = 5), sodium selenite and vitamin E (black triangles solid lines; SG, *n* = 5), arsenite + sodium selenite and vitamin E (black circles lines dashed; ASG, *n* = 5), and physiological solution (black circles dashed lines; CG, *n* = 5), for 12 weeks. ^a, b^ Values with different literal difference (*p* ≤ 0.05). Mean values (±SEM).

Table 2 shows the values for the physiological constants of male goats; differences among treatments and time occurred (*p* ≤ 0.05) for heart and respiratory rate (Figure 3). The ASG and SG groups showed a lower heart rate compared to the CG and AG groups (58.8 ± 11.5, 58 ± 9.6 vs. 65.4 ± 10.4, 63.5 ± 9.4, respectively; *p* ≤ 0.05). Similarly, the SG and CG showed a lower respiratory rate than the AG and ASG (21.0 ± 5.8, 22.1 ± 4.7, vs. 19.2 ± 1.9, 18.7 ± 2.6, respectively; *p* ≤ 0.05).

**Figure 4:**
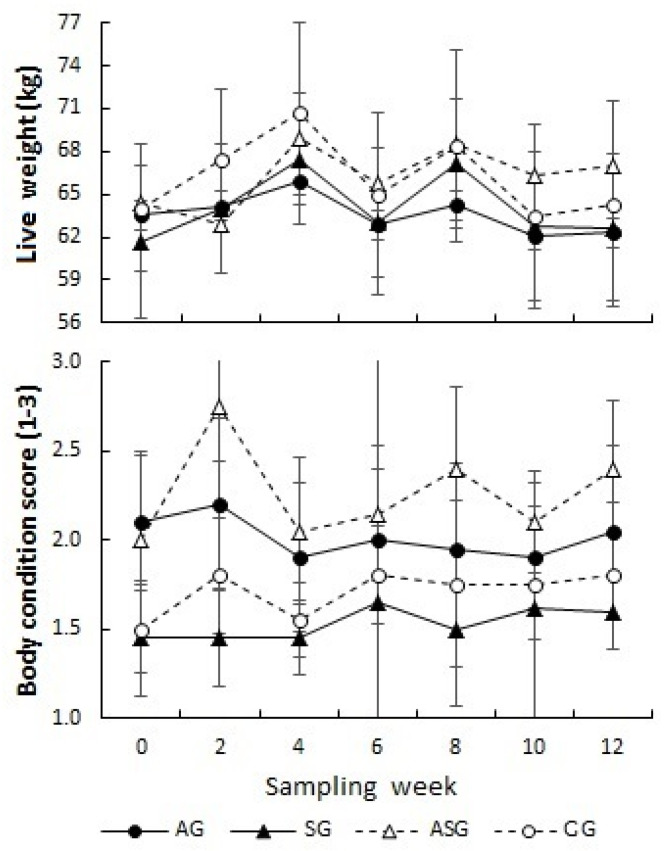
Across time of body parameters of male goats treated with sodium arsenite (black circles solid lines; AG, *n* = 5), sodium selenite and vitamin E (black triangles solid lines; SG, *n* = 5), arsenite + sodium selenite and vitamin E (black circles lines dashed; ASG, *n* = 5), and physiological solution (black circles dashed lines; CG, *n* = 5), for 12 weeks. Mean values (±SEM).

Table 3 shows the results of the body parameters of the male goats. There were no significant differences between live weight (65.05 ± 5.9) and body condition (2.05 ± 1.4) among the groups for treatment effects (*p* > 0.05). However, differences in body condition across time can be observed (Figure 4); the ASG group maintained the highest levels (2.26 ± 0.38) without showing a statistical difference with the AG and CG (2.26 ± 0.38, 2.01 ± 0.41 and 1.71 ± 0.32; respectively, *p* > 0.05), but there was a difference compared to the SG (2.26 ± 0.38 vs. 1.53 ± 0.53; respectively, *p* ≤ 0.05).

## 4. Discussion

We hypothesized that the antioxidant potential of selenium and vitamin-E would counteract the toxic effects caused by the consumption of water contaminated with sodium arsenite, which is reflected in reproductive disorders and alteration of physiological constants in Criollo male goats of the Comarca Lagunera. The results show that sodium arsenite does affect sperm quality; the effects have been mainly visible in the viability variable, considered one of the main fertility markers in males [32]. In contrast, the SG group obtained the highest percentage of live sperms together with the ASG and CG groups; previous studies also generated similar results with selenium and vitamin-E supplementation [11,33]. Arsenic-mediated toxicity in male reproduction is considered to be related to spermatotoxicity [34], caused by the production of reactive oxygen species (ROS) [35,36]; excessive levels of ROS can negatively affect sperm quality by decreasing motility while increasing DNA damage and lipid peroxidation of the cellular membrane [33]. Therefore, motility was probably affected at week 8 in the AG group; it decreased from a value of 2.5 (good motility) to 1.0 (no motility) by the end of the study. These results are consistent with research carried out on murine and caprine species [11,37].

Regarding the volume, we can observe that the males treated with arsenic across time obtained the lowest values, except in week 4. However, at the end of the treatment period, the males of the ASG group obtained the highest volume of ejaculation; these results agree with Zubair et al. (2016) who report that in animals treated with arsenic the semen volume is significantly reduced, from week 6 to week 12 of treatment. Zubair attributed such decreases in volume to the reduction in the level of testosterone due to the toxicity of arsenic [11]. Souza et al. (2015) reported that sodium arsenite not only reduces the percentage of seminiferous epithelium, its volume, and the proportion of Leydig cells, but also decreases the testicular antioxidant-defense activity [38]. On the contrary, the ASG group showed an increase in the seminal volume, probably because selenium operated in the body of the male goats through selenoproteins, which act directly on the testes through glutathione peroxidase (GPX), an antioxidant enzyme that ensures the viability of sperm [19]. Similarly, previous research has shown that vitamin-E has synergistic effects with selenium. Vitamin-E ensures the proper function of the epithelium in the seminiferous tubules [39].

In this study, the dose of sodium arsenite used did not generate significant differences in sperm concentration in the AG group; however, other studies testing higher doses (5, 10 and 100 mg/kg) have reported decreased sperm concentration in male Wistar rats and male goats [11,33,38]. On the other hand, in the selenium (SG)-supplemented group, a very favorable numerical response was obtained, both in the color parameter and sperm concentration, which significantly increased its concentration from week 6. The latter is in agreement with Ahmadi et al. (2016) and Zubair et al. (2017) who mention that selenium and vitamin-E improve sperm concentration, through selenoproteins, mainly GPX [39,40]. Along with selenium, vitamin-E interrupts the reactions with lipid peroxidation; both prevent phospholipid peroxidation in the sperm mitochondria and, therefore, their maximum immotility [41].

At week 12, the odor of males treated with selenium SG increased, coinciding with the most important variables characterized within the fertility parameters in males (i.e., viability, motility and sperm concentration). The reason for this improvement may be due to the contribution of selenium and vitamin-E in the production and maturation of sperm, in addition to the fact that both have been documented to be required for testosterone biosynthesis [40,42]. Certainly, when testosterone concentrations increase, odor also increases; such odor-pheromones occur in the sebaceous glands and contribute to the male effect [43]. Furthermore, in addition to the toxicity caused by arsenic in the male reproductive system [11,38], arsenic causes cardiac and vascular effects [25,44]. Our results confirm that fact because the highest heart rate occurred in the AG group; the reason for this may be the oxidative stress caused by arsenic, which leads to damage to the endothelial cells causing greater platelet adhesion and reduction in vasodilation; such responses could be the main mechanism of hypertension [45]. Indeed, arsenic toxicity promotes cardiac and vascular events such as misfunction of myocardial and vascular endothelial cells, vasoconstriction, and an increased susceptibility to platelet aggregation [25]. Conversely, in males treated with selenium and vitamin-E, the heart rate was the lowest, probably due to the antioxidant elements, which exerted their functions through selenoproteins which are essential for the proper function of the cardiovascular system, which generated a drop in heart rate [46].

On the other hand, the AG group had a higher respiratory rate than the CG group; according to Ahamed et al. (2006), chronic arsenic poisoning promotes the appearance of such symptoms from week two to eight [47]. Biswas et al. (2000) reported that until week nine, they observed an increase in the respiratory rate in goats exposed to 25 mg^−1^ of arsenic, concluding that such a response could be due to the retention of toxic metabolites resulting from acidosis [44]. Interestingly, in the SG group, the respiratory rate was not altered, suggesting that selenium and vitamin-E probably depressed the toxic effects of arsenic, due to their strong antioxidant property [11,48,49]. Previously Chauhan et al. (2015), demonstrated that dietary supplementation with selenium or vitamin-E individually reduced the negative effects of the respiratory rate in sheep subjected to heat stress [50]. These authors demonstrated that combined supplementation (selenium + vitamin-E) resulted in a synergistic action to prevent the oxidative damage of cellular macromolecules such as proteins by reducing the oxidative stress index, as well as increasing the expression and activity of antioxidant enzymes.

## 5. Conclusions

In conclusion, the results of this study demonstrate that treatment with selenium and vitamin-E, due to its antioxidant potential, reduced the damage caused by sodium arsenite in sperm quality, increased odor and maintained heart and respiratory rates in Criollo male goats from the Comarca Lagunera, a semiarid region in northern Mexico. The information derived from the present investigation is a reference to propose the use of these antioxidants to reduce the damage caused by arsenic in the animal organisms in places where groundwater contaminated with arsenic is used as drinking water for animals; a common scenario seen under arid and semiarid environmental conditions.

## Figures and Tables

**Table 1:** Reproductive parameters of male goats treated with sodium arsenite (AG), sodium selenite and vitamin-E (SG), arsenite + sodium selenite and vitamin-E (ASG) and physiological solution (CG) for 12 weeks.

Reproductive Parameters	AG (*n* = 5)	SG (*n* = 5)	ASG (*n* = 5)	CG (*n* = 5)	*p*-Value		
					Treatment	Time	Treatment × Time
Scrotal circumference (cm)	28.4 ± 1.3	30.9 ± 3.2	30.1 ± 1.7	29.6 ± 1.8	0.281	0.201	0.740
Odor (1–3)	1.6 ± 0.4	1.5 ± 0.4	1.6 ± 0.5	1.6 ± 0.4	0.876	0.001	0.122
Latency (s)	33.3 ± 21.9	35.0 ± 27.3	27.3 ± 26.1	24.1 ± 20.7	0.593	0.310	0.092
Volume (mL)	0.8 ± 0.4	0.9 ± 0.4	1.1 ± 0.4	1.0 ± 0.5	0.210	0.025	0.111
Viability (%)	64.3 ± 29.3 ^b^	80.6 ± 19.9 ^a^	71.9 ± 18.3 ^ab^	77.5 ± 17.0 ^ab^	0.003	0.002	0.557
Motility (1–3)	2.1 ± 0.8	2.3 ± 0.4	2.1 ± 0.7	2.3 ± 0.6	0.565	0.006	0.001
Concentration (Cell × 10^6^ mL)	4344.6 ± 251.4	3970.7 ± 1007.8	4491.4 ± 179.2	4377.7 ± 522.4	0.800	0.475	0.426
Color (1–3)	1.5 ± 0.5	1.9 ± 0.8	2.2 ± 0.8	1.9 ± 0.78	0.235	0.050	0.606

^a,b^ = Values with different literal difference (*p* ≤ 0.05); mean values (±SEM).

**Table 2:** Physiological constants of male goats treated with sodium arsenite (AG), sodium selenite and vitamin-E (SG), arsenite + sodium selenite and vitamin-E (ASG) and physiological solution (CG) for 12 weeks.

Physiological Constants	AG (*n* = 5)	SG (*n* = 5)	ASG (*n* = 5)	CG (*n* = 5)	*p*-Value		
					Treatment	Time	Treatment × Time
Heart rate (bpm)	63.5 ± 9.4 ^a^	58 ± 9.6 ^b^	58.8 ± 11.5 ^b^	65.4 ± 10.4 ^a^	0.050	0.029	0.073
Respiratory rate (rpm)	22.1 ± 4.7 ^a^	19.2 ± 1.9 ^ab^	21.0 ± 5.8 ^ab^	18.7 ± 2.6 ^b^	0.038	0.050	0.355
Rumen movement (1–3)	1.9 ± 0.6	1.8 ± 0.5	1.7 ± 0.6	1.9 ± 0.6	0.973	0.187	0.830
Body temperature (°C)	38.5 ± 0.8	38.6 ± 0.4	38.8 ± 0.6	38.5 ± 0.6	0.164	0.367	0.357

^a,b^ = Values with different literal difference (*p* ≤ 0.05). Mean values (±SEM).

**Table 3:** Body parameters of male goats treated with sodium arsenite (AG), sodium selenite and vitamin-E (SG), arsenite + sodium selenite and vitamin-E (ASG) and physiological solution (CG) for 12 weeks.

Body Parameters	AG (*n* = 5)	SG (*n* = 5)	ASG (*n* = 5)	CG (*n* = 5)	*p*-Value		
					Treatment	Time	Treatment × Time
LW (Kg)	63.6 ± 4.9	64.1 ± 5.0	66.3 ± 7.7	66.2 ± 6.1	0.839	0.08	0.086
BCS (1–3)	2.0 ± 0.4	2.2 ± 4.3	2.3 ± 0.4	1.7 ± 0.3	0.703	0.025	0.830

LW = Live weight; BCS = Body condition score. Mean values (±SEM).

## Data Availability

The corresponding author can provide access to the datasets upon request.
